# Identification of biomarkers for Barcelona Clinic Liver Cancer staging and overall survival of patients with hepatocellular carcinoma

**DOI:** 10.1371/journal.pone.0202763

**Published:** 2018-08-23

**Authors:** Wei Xu, Quan Rao, Yongbo An, Mengyi Li, Zhongtao Zhang

**Affiliations:** Department of General Surgery, Beijing Friendship Hospital, Capital Medical University, Beijing Key Laboratory of Cancer Invasion and Metastasis Research, National Clinical Research Center for Digestive Diseases, Beijing, P.R. China; Centre de Recherche en Cancerologie de Lyon, FRANCE

## Abstract

The aim of the current study was to identify biomarkers that correlate with the Barcelona Clinic Liver Cancer (BCLC) staging system and prognosis of patients with hepatocellular carcinoma (HCC). We downloaded 4 gene expression datasets from the Gene Expression Omnibus database (http://www.ncbi.nlm.nih.gov/geo), and screened for genes that were differentially expressed between HCC and normal liver tissues, using significance analysis of the microarray algorithm. We used a weighted gene co-expression network analysis (WGCNA) to identify hub genes that correlate with BCLC staging, functional enrichment analysis to associate hub genes with their functions, protein-protein interaction network analysis to identify interactions among hub genes, UALCAN analysis to assess gene expression levels based on tumour stage, and survival analyses to clarify the effects of hub genes on patients’ overall survival (OS). We identified 50 relevant hub genes using WGCNA; among them, 13 genes (including *TIGD5*, *C8ORF33*, *NUDCD1*, *INSB8*, and *STIP1*) correlated with OS and BCLC staging. Significantly enriched gene ontology biological process terms included RNA processing, non-coding RNA processing and phosphodiester bond hydrolysis, and 6 genes were found to interact with 10 or more hub genes. We identified several candidate biomarkers that correlate with BCLC staging and OS of HCC. These genes might be used for prognostic assessment and selection of HCC patients for surgery, especially those with intermediate or advanced disease.

## Introduction

Hepatocellular carcinoma (HCC) ranks fifth in cancer incidence and third in cancer mortality worldwide [1]. Barcelona Clinic Liver Cancer (BCLC) staging is a commonly used staging system for HCC that considers tumour burden, liver function, and patients’ general condition; for patients with early-stage HCC, it also analyses indications for hepatic resection [2]. However, increasing evidence indicates that patients with intermediate or advanced HCC might benefit from hepatic resection [3–9]. Our previous study showed that prognoses of patients at the same BCLC stage varied significantly after surgery. For instance, among patients in BCLC stage B, 14.7% died within 1 year after surgery whereas 33.8% were still alive after 5 years [10]. We conclude that HCC is a highly heterogeneous and comprehensive tumour even within the same clinical stage.

Differences in prognosis after hepatectomy for HCC might result from different biological behaviours of tumours, which could be assessed by different expression levels of genes correlated with BCLC staging and patients’ overall survival (OS). Thus, identification of differentially expressed genes (DEGs) in HCC is clinically significant, as their products could be markers for survival, prognostic effects of liver resection, or surgical indication, especially among patients with intermediate or advanced HCC. Other studies have used microarray technology to identify HCC-associated DEGs in daily clinical practice, including applications for differential diagnosis, risk assessment, determination of prognosis, disease monitoring and prediction of treatment response [11–13]. For instance, Sakabe T et al. reported that DKK1 might be an unfavourable prognostic marker for HCC patients [11]; Zheng H et al. found that TMED3 promotes HCC progression [12]; and Emma MR et al. identified NUPR1 as a promising target in HCC for its effects in controlling cell growth, migration, invasion and sorafenib resistance [13]. However, their respective gene expression datasets were not fully utilized due to restricted purposes or limited tools, and information on potential biomarkers associated with BCLC staging and patients’ prognosis remains scarce.

In the present study we identified HCC-associated DEGs through differential analysis of 3 gene expression datasets. Another dataset from surgical specimens was used to perform a weighted gene co-expression network analysis (WGCNA) for upregulated DEGs. Gene modules with close relationships to the BCLC staging system were screened. We also used UALCAN analysis, an online tool based on The Cancer Genome Atlas (TCGA) datasets, to reassess the expression levels of hub genes associated with tumour stages and OS.

## Methods

### Gene expression data

We downloaded 4 gene expression datasets from the gene expression omnibus (GEO, http://www.ncbi.nlm.nih.gov/geo/) database [14]. GSE87630 included 64 HCC tissues and 30 non-tumour liver tissues [15], and used Illumina HumanHT-12 V3.0 expression beadchip (Illumina Inc., San Diego, California, USA). GSE84598 analysed 22 pairs of HCC and normal liver tissues using the Illumina HumanHT-12 V4.0 expression beadchip (Illumina) [16]. GSE45267 contained 48 primary HCC samples, and 39 non-cancerous samples, from 61 patients [17], and used Affymetrix Human Genome U133 Plus 2.0 Array (Affymetrix Inc., Santa Clara, California, USA). GSE87630, GSE84598, and GSE45267 were used to identify DEGs between HCC and normal liver tissue. GSE76427 included HCC samples from 115 patients, based on Illumina HumanHT-12 V4.0 expression beadchip arrays, with clinicopathological data that included sex, age, BCLC stage, tumour recurrence, and OS after surgeries and was used to identify possible relationships between DEGs and clinical traits [18].

The raw data were pre-processed. Probes were matched to genes and gene expression levels were taken as the average probe values for genes corresponding to more than one probe. Log2 conversion and quantile normalization were applied to data if appropriate. Genes with more than 20% missing values were removed.

### Identification of DEGs

The Limma package in Bioconductor was used to analyse DEGs in HCC tissues compared with normal liver samples. *P* values of DEGs were calculated using Limma package of R. |Log2FC|≥1; P<0.05 was considered significant.

### Functional enrichment analysis

Gene ontology (GO) is a major bioinformatics tool for gene annotation that uses a highly structured vocabulary including three main categories: molecular functions (MF), biological processes (BP) and cellular components (CC) [19]. The Kyoto Encyclopaedia of Genes and Genomes (KEGG) is a database used to associate related genes by pathways [20]. The Database for Annotation, Visualization, and Integrated Discovery (DAVID; http://david.ncifcrf.gov; version 6.8) provides a comprehensive set of functional annotation tools that enables investigators to understand the biological meaning behind large lists of genes [21]. In the present study, DAVID online tools were used to perform GO annotation and KEGG pathway enrichment analyses of DEGs. P<0.05 and false discovery rate (FDR) <0.25 were set as thresholds.

### WGCNA

A gene co-expression network was constructed to screen for genes related to BCLC staging among the DEGs. The WGCNA package of R was used [22]. Before network construction, obvious outlier samples or samples with excessive numbers of missing entries were removed using a sample network method for outlier detection. Step-by-step network construction and module detection were performed [22]. The soft-threshold power was selected based on the criterion of approximate scale-free topology. We calculated adjacencies using soft-threshold power and then transformed the adjacency into Topological Overlap Matrix (TOM) and calculated the corresponding dissimilarities. Average linkage hierarchical clustering was performed based on the TOM with a minimum module size of 30 and a medium sensitivity of 2, and gene modules with very similar expression probes were identified and merged. Furthermore, after correlating gene modules with external clinical traits, the associated gene significance (GS, the absolute value of the correlation between the gene and the trait) and module membership (MM, the correlation of the module gene and the gene expression profile) were assessed. Based on GS and MM, specific gene modules related to BCLC staging were selected and the top 50 core genes in the selected modules were screened and regarded as hub genes.

### Functional annotation analysis for hub genes

To analyse hub gene functions at the molecular level, GO functional enrichment and KEGG pathway analysis were performed using the DAVID online tool. The Search Tool for the Retrieval of Interacting Genes (STRING; http://string-db.org; version 10.5) database was used to construct a protein–protein interaction (PPI) network [23]. An interaction with a combined score >0.4 was considered statistically significant.

### UALCAN analysis

UALCAN is an interactive web-portal for facilitating tumour subgroup gene expression and survival analyses (http://ualcan.path.uab.edu/analysis.html) [24]. We used UALCAN analysis to estimate the effects of hub gene expression levels based on tumour stages in the Cancer Genome Atlas (TCGA) liver cancer datasets. Available TCGA patient survival data were also used for Kaplan–Meier survival analyses.

## Results

### Identification of DEGs

Sets of 19611, 20258 and 22185 genes were detected in the datasets of GSE87630, GSE84598, and GSE45267, respectively, after pre-recession of raw data. DEGs were screened in 3 datasets, which contained both tumour and normal tissues. Cohorts of 1163, 1914, and 1582 DEGs were obtained in GSE87630, GSE84598, and GSE45267, respectively. Only 352 genes were common among the 3 sets of DEGs; 623 genes were common between 2 sets of DEGs; and 2340 genes were unique to one set. This suggests a high heterogeneity among HCC samples. The combined 3 sets of DEGs, a total of 3315 genes, of which 1573 were upregulated and 1748 were downregulated, were regarded as HCC-related DEGs for further analysis.

### Functional enrichment analysis

Results of GO analysis showed that upregulated DEGs were significantly enriched in cell division, sister chromatid cohesion, and DNA replication in BP; protein binding, poly-A RNA binding and ATP binding in MF; and nucleoplasm, nucleus and cytosol in CC. Among downregulated DEGs, significantly enriched GO-terms included oxidation–reduction process, immune response and xenobiotic metabolic process in BP; haem binding, monooxygenase activity and oxygen binding in MF; and extracellular space, extracellular region and extracellular exosome in CC (**Table 1**).

**Table 1:** Gene ontology terms that were significantly enriched in differentially expressed genes.

Term	Description	Count in gene set	%	P-value	FDR^#^
Up-regulated					
GOTERM_BP	GO:0051301~cell division	100	6.37	1.72E-29	3.18E-26
GOTERM_BP	GO:0007062~sister chromatid cohesion	45	2.86	1.28E-21	2.37E-18
GOTERM_BP	GO:0006260~DNA replication	55	3.50	3.17E-21	5.86E-18
GOTERM_BP	GO:0007067~mitotic nuclear division	70	4.46	1.81E-20	3.35E-17
GOTERM_BP	GO:0006270~DNA replication initiation	20	1.27	2.22E-13	4.10E-10
GOTERM_MF	GO:0005515~protein binding	914	58.18	1.35E-29	2.20E-26
GOTERM_MF	GO:0044822~poly A RNA binding	152	9.68	4.29E-10	6.97E-07
GOTERM_MF	GO:0005524~ATP binding	174	11.08	8.93E-07	1.45E-07
GOTERM_MF	GO:0003682~chromatin binding	58	3.69	1.20E-05	0.02
GOTERM_CC	GO:0005654~nucleoplasm	415	26.42	8.37E-42	1.27E-38
GOTERM_CC	GO:0005634~nucleus	595	37.87	6.10E-22	9.26E-19
GOTERM_CC	GO:0005829~cytosol	390	24.82	6.99E-18	1.06E-14
GOTERM_CC	GO:0000777~condensed chromosome kinetochore	35	2.23	7.90E-16	1.18E-12
GOTERM_CC	GO:0005737~cytoplasm	550	35.01	2.27E-15	3.36E-12
Down-regulated					
GOTERM_BP	GO:0055114~oxidation-reduction process	123	7.48	7.39E-21	1.40E-17
GOTERM_BP	GO:0006955~immune response	95	5.78	1.03E-18	1.96E-15
GOTERM_BP	GO:0006805~xenobiotic metabolic process	33	2.01	7.02E-15	1.33E-11
GOTERM_BP	GO:0002576~platelet degranulation	38	2.31	8.74E-15	1.66E-11
GOTERM_BP	GO:0006954~inflammatory response	80	4.87	4.79E-14	9.10E-11
GOTERM_MF	GO:0020037~heme binding	52	3.16	5.52E-21	9.29E-18
GOTERM_MF	GO:0004497~monooxygenase activity	31	1.89	1.07E-17	1.81E-14
GOTERM_MF	GO:0019825~oxygen binding	27	1.64	1.82E-16	3.77E-13
GOTERM_MF	GO:0005506~iron ion binding	48	2.92	1.35E-15	2.24E-12
GOTERM_MF	GO:0016491~oxidoreductase activity	52	3.16	3.41E-13	5.74E-10
GOTERM_CC	GO:0005615~extracellular space	247	15.02	1.29E-34	1.89E-31
GOTERM_CC	GO:0005576~extracellular region	278	16.91	1.92E-34	2.82E-31
GOTERM_CC	GO:0070062~extracellular exosome	406	24.70	5.59E-33	8.22E-30
GOTERM_CC	GO:0072562~blood microparticle	57	3.47	6.90E-23	1.01E-19
GOTERM_CC	GO:0031093~platelet alpha granule lumen	29	1.76	1.66E-16	1.67E-13

^#^FDR: false discovery rate

The KEGG pathways of the upregulated DEGs were enriched in cell cycle, DNA replication, and viral carcinogenesis; and those of the downregulated DEGs were enriched in complement and coagulation cascades, metabolic pathways, chemical carcinogenesis, and drug metabolism by cytochrome P450 (Table 2).

**Table 2:** Kyoto Encyclopaedia of Genes and Genomes pathways that were significantly enriched in the differentially expressed genes.

Expression	Description	Count in gene set	%	P-value	FDR^#^
Up-regulated	hsa04110:Cell cycle	41	2.61	5.37E-14	7.09E-11
	hsa03030:DNA replication	19	1.21	1.26E-10	1.66E-07
	hsa05203:Viral carcinogenesis	38	2.42	1.19E-05	1.58E-02
Down-regulated	hsa04610:Complement and coagulation cascades	39	2.37	3.52E-19	4.67E-16
	hsa01100:Metabolic pathways	222	13.50	1.62E-15	2.21E-12
	hsa05204:Chemical carcinogenesis	35	2.13	6.23E-13	8.25E-10
	hsa00982:Drug metabolism—cytochrome P450	31	1.89	4.54E-12	6.01E-09
	hsa00071:Fatty acid degradation	24	1.46	1.91E-11	2.53E-08

^#^FDR: false discovery rate

### WGCNA

**Fig 1:**
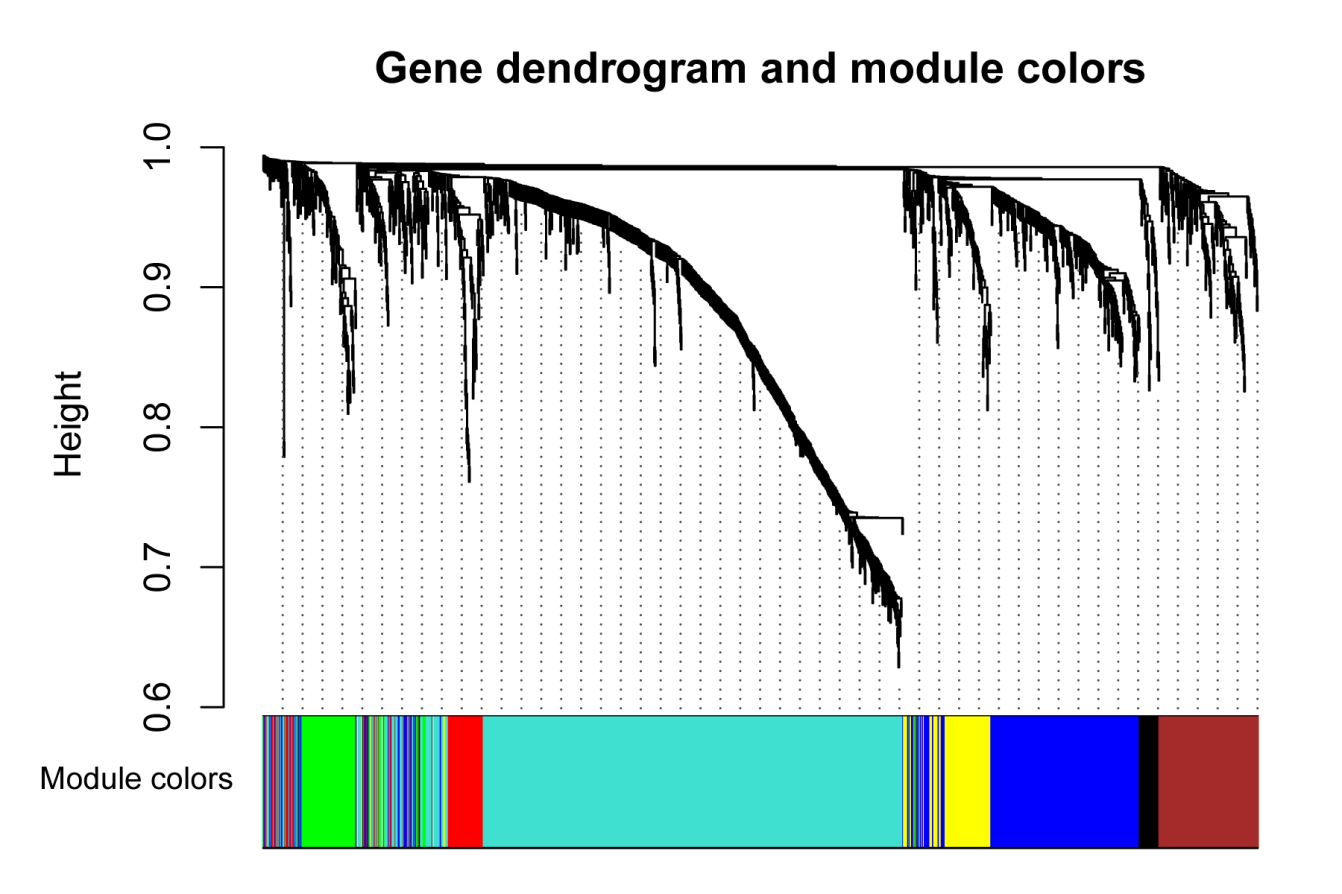
Clustering dendrogram of genes. Clustering dendrogram of genes, with dissimilarity based on topological overlap, together with assigned module colours.

**Fig 2:**
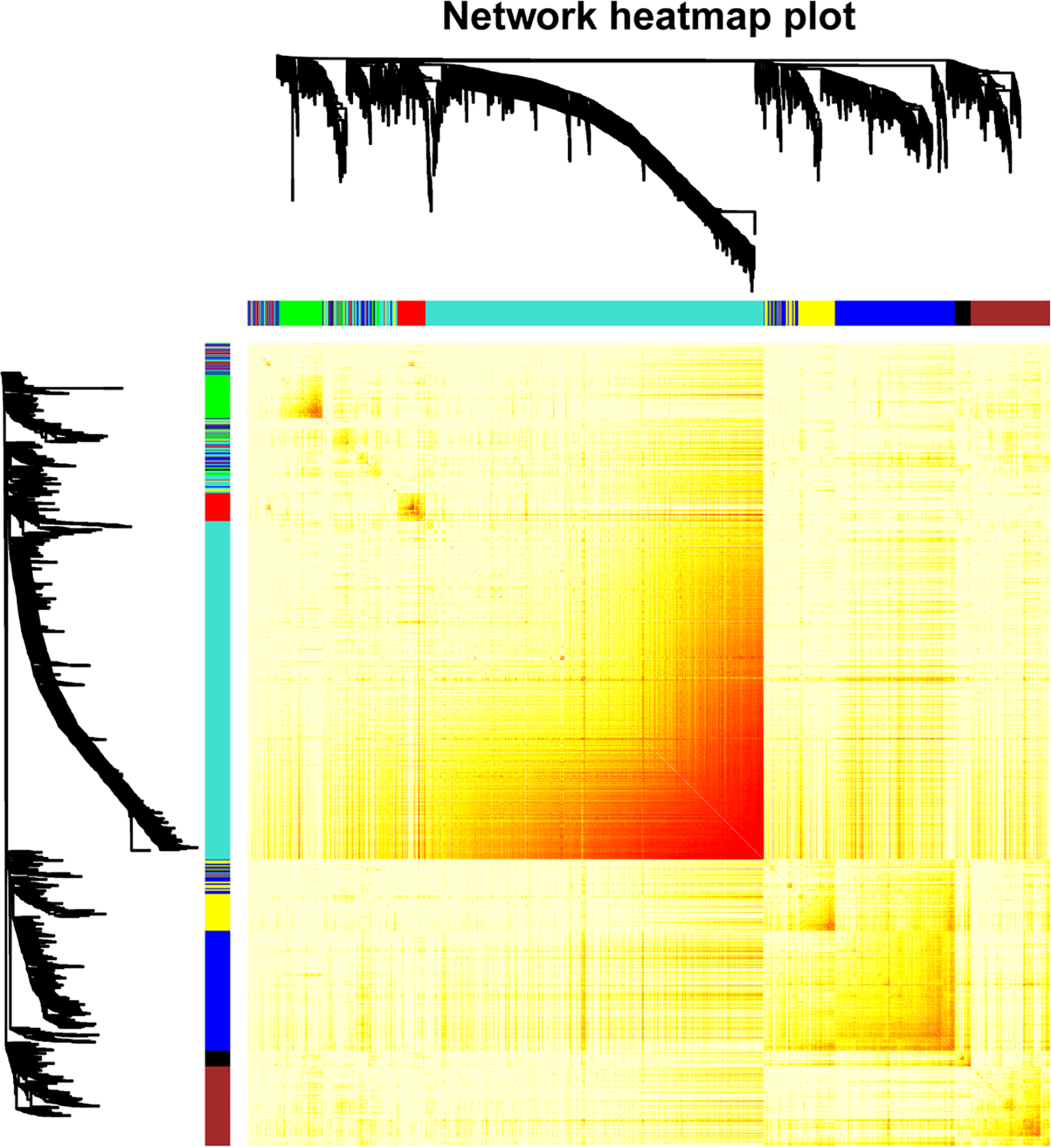
Heat map of genes. The heat map depicts the Topological Overlap Matrix among genes in the analysis. Genes in modules such as Green and Turquoise with high overlap are shown in dark red.

To further screen relationships between the 1573 upregulated DEGs and clinical traits, we performed WGCNA. Among 1573 DEGs, 1568 gene expressions mapped to probes in GSE76427 (S1 Table), which contained pre-processed raw data. Based on WGCNA results, systematic clustering of upregulated DEGs was generated (Fig 1). The gene modules were signified by different colours. According to the hierarchical clustering tree of genes, 7 gene modules were identified with the soft-threshold power of 3 and the gene number of gene modules ranged from 41 (black) to 756 (turquoise). The heat map of upregulated DEGs classified by gene modules was shown in Fig 2.

**Fig 3:**
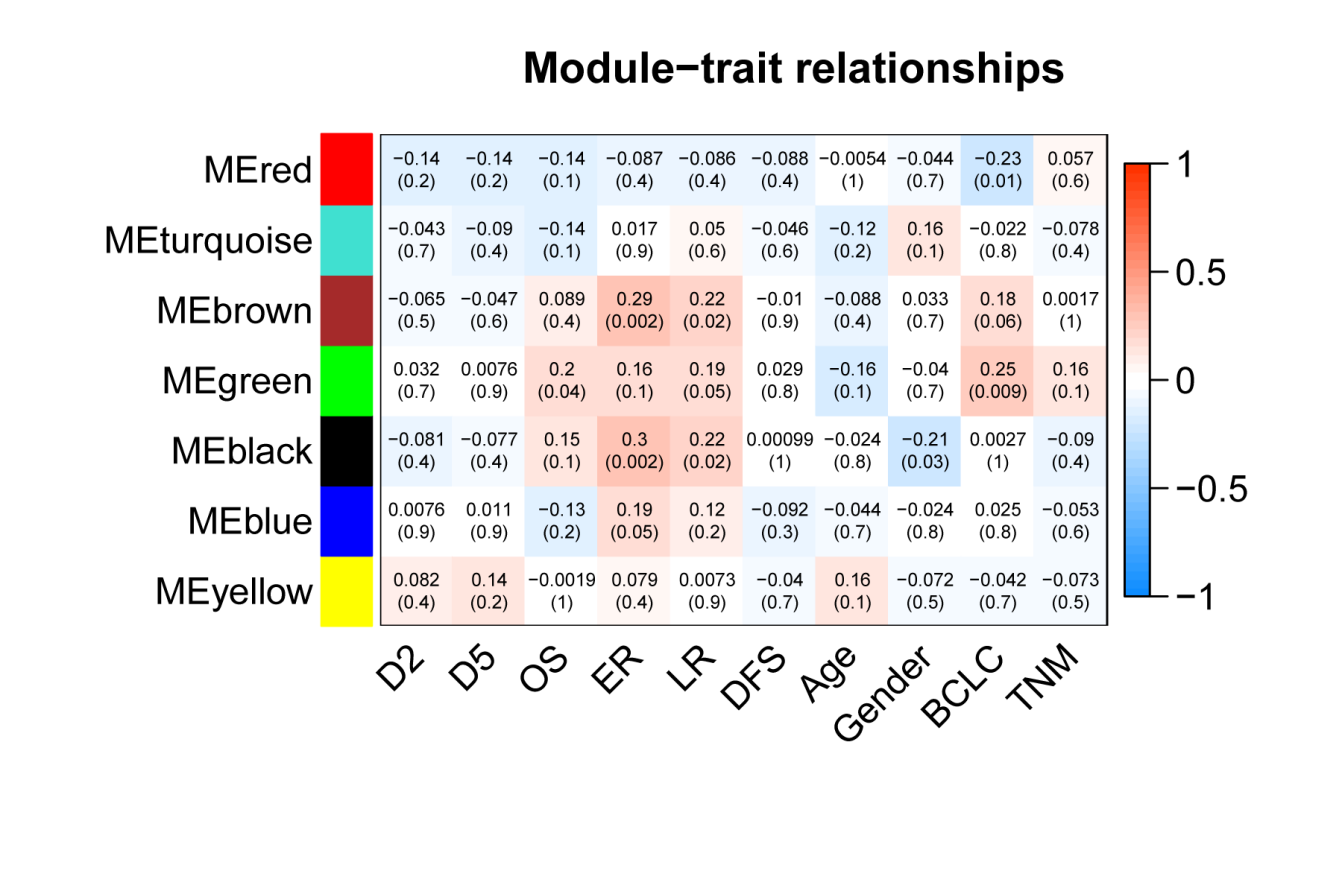
Module−trait relationships. Genes in the Green module showed a closer relationship with BCLC staging than other gene modules and were also correlated with overall survival.

The gene modules were also associated with external clinical traits (S2 Table) (Fig 3). Genes in the Green module clearly showed a closer relationship with BCLC staging than any other gene modules, and were also correlated with OS. Based on GS and MM, the top 50 core genes in the Green module were regarded as hub genes, including *PUF60*, *BOP1*, and *SCRIB* (S3 Table).

### Functional annotation analysis for hub genes

Results of GO analysis showed that hub genes were mainly concentrated in RNA processing, non-coding RNA processing and RNA phosphodiester bond hydrolysis in BP; RNA binding, nucleic acid binding and poly-A-RNA binding in MF; and nucleus, nuclear lumen and nuclear part in CC (Table 3). No other significantly enriched pathways of hub genes were identified. Based on the information in the STRING databases, we created the PPI network of hub genes. *HSP90AB1*, *BOP1*, *DCAF13*, *RPL8* and *STIP1* were the 5 genes with the most interactions with other hub genes, and were at the core of the PPI network.

**Table 3:** Gene ontology terms that were significantly enriched in hub genes.

Term	Description	Count in gene set	%	P-value	FDR^#^
GOTERM_BP	GO:0006396~RNA processing	10	ZC3H3, DCAF13, INTS8, PUSL1, RPL8, POP1, AGO2, RRS1, BOP1, PUF60	1.52E-04	0.24
GOTERM_BP	GO:0034470~ncRNA processing	8	DCAF13, INTS8, PUSL1, RPL8, POP1, AGO2, RRS1, BOP1	3.28E-05	0.05
GOTERM_BP	GO:0090502~RNA phosphodiester bond hydrolysis, endonucleolytic	5	AGO2, BOP1, POP1,RRS1, ZC3H3	1.94E-05	0.03
GOTERM_MF	GO:0003723~RNA binding	16	HSP90AB1, PUSL1, PRKDC, STIP1, BOP1, STAU2, DCAF13, UBR5, RPL8, POP1…	2.64E-06	3.34E-03
GOTERM_MF	GO:0003676~nucleic acid binding	23	BOP1, DSCC1, DCAF13, HSPB1, ZC3H3, HSP90AB1, ZNF623, PUSL1, ZHX1, PRKDC…	2.91E-05	0.04
GOTERM_MF	GO:0044822~poly(A) RNA binding	12	HSP90AB1, DCAF13, RPL8, POP1, AGO2, RRS1, HSPB1, PRKDC, STIP1, BOP1…	7.35E-05	0.09
GOTERM_CC	GO:0005634~nucleus	32	ZC3H3, HSP90AB1, GSDMD, ZNF250, PRKDC, STIP1, BOP1, UBD,ZNF16, STAU2 …	1.65E-06	2.09E-03
GOTERM_CC	GO:0031981~nuclear lumen	21	ZC3H3, HSP90AB1, GSDMD, ZHX1, PRKDC, BOP1, TOP1MT, TATDN1, SCRIB, STAU2…	2.69E-05	0.03
GOTERM_CC	GO:0044428~nuclear part	21	ZC3H3, HSP90AB1, GSDMD, ZHX1, PRKDC, BOP1, TOP1MT, TATDN1, SCRIB, STAU2…	1.25E-04	0.16

^#^FDR: false discovery rate

### UALCAN analysis

Among the 50 hub genes, 45 were identified from TGCA HCC database on UALCAN; all 45 showed significantly higher expression levels in primary tumour tissue than normal liver tissue. Furthermore, 18 hub genes (*TIGD5*, *C8ORF33*, *ZNF250*, *NUDCD1*, *INTS8*, *ZNF623*, *PHF20L1*, *STIP1*, *ZNF16*, *HSP90AB1*, *LRRC14*, *DSCC1*, *POP1*, *ARHGAP39*, *PRKDC*, *YDJC*, *PUSL1*, *UBD*) showed significantly different expression levels by different tumour stages. In survival analysis, lower expression of 24 hub genes (*BOP1*, *TIGD5*, *C8orf76*, *C8orf33*, *FAM83H*, *UBR5*, *NSMCE2*, *DCAF13*, *PYCRL*, *NUDCD1*, *INTS8*, *ZNF623*, *TOP1MT*, *STIP1*, *HSP90AB1*, *PRR7*, *COMMD5*, *DSCC1*, *POP1*, *ARHGAP39*, *PRKDC*, *YDJC*, *PUSL1*, *STAU2*) was associated with significantly longer OS of HCC patients. Based on UALCAN analysis, 13 hub genes (*TIGD5*, *C8ORF33*, *NUDCD1*, *INTS8*, *ZNF623*, *STIP1*, *HSP90AB1*, *DSCC1*, *POP1*, *ARHGAP39*, *PRKDC*, *YDJC* and *PUSL1*) were significantly correlated with BCLC staging and OS (Table 4).

**Table 4:** Expression level of hub genes in each tumour stage and effect of gene expression level on HCC patients’ survival.

Hub genes	Median Expression (Transcript per million)					*P*-value	Median survival time (month)		*P*-value
	Stage 0	Stage A	Stage B	Stage C	Stage D		High expression	Low/Medium expression	
TIGD5	1.17 (0.52–1.97)	4.30 (1.22–14.32)	5.09 (0.76–13.08)	5.73 (1.13–16.03)	4.57 (1.85–6.03)	<0.01	46.0	62.0	0.01
C8ORF33	7.46 (4.63–11.30)	23.03 (6.85–64.67)	24.84 (8.11–70.03)	26.60 (7.66–60.94)	32.74 (15.56–65.50)	<0.01	42.6	70.8	<0.01
NUDCD1	1.79 (0.44–2.69)	3.84 (0.56–10.74)	4.08 (0.57–10.65)	4.40 (1.14–14.93)	5.08 (1.17–7.68)	<0.01	25.6	62.0	<0.01
INTS8	2.87 (1.16–5.34)	7.66 (1.81–21.59)	8.78 (3.23–23.46)	9.13 (2.71–31.92)	6.33 (3.34–14.44)	<0.01	38.4	70.8	<0.01
ZNF623	1.17 (0.20–2.23)	2.57(0.25–8.51)	2.62(0.51–8.65)	3.51(0.52–10.94)	2.20(0.61–4.84)	<0.01	42.6	56.7	0.05
STIP1	22.93 (12.94–33.80)	56.86 (10.60–130.54)	65.24 (23.77–165.35)	76.67 (20.08–204.91)	64.03 (33.23–91.54)	<0.01	22.1	71.3	<0.01
HSP90AB1	196.33 (121.63–281.73)	505.67 (99.54–1266.42)	592.33 (207.77–1449.43)	684.23 (26.83–1662.28)	759.80 (297.47–1101.89)	<0.01	30.0	62.0	<0.01
DSCC1	0.26 (0.04–0.63)	1.49 (0.09–5.36)	1.67 (0.29–5.99)	2.16 (0.20–7.05)	1.55 (0.71–4.36)	<0.01	28.0	62.1	<0.01
POP1	0.47 (0.23–0.94)	1.01 (0.26–2.49)	1.20 (0.32–2.98)	1.26 (0.28–3.28)	1.00 (0.56–2.24)	<0.01	25.7	62.0	<0.01
ARHGAP39	0.20 (0.06–0.36)	0.67 (0.09–2.18)	0.89 (0.10–2.49)	0.94 (0.07–4.00)	0.71 (0.12–1.59)	<0.01	33.6	71.2	<0.01
PRKDC	3.13 (0.85–5.74)	6.74 (0.85–22.28)	7.83 (0.95–30.85)	9.09 (0.46–35.24)	5.10 (2.90–6.70)	<0.01	42.5	56.5	0.02
YDJC	5.59 (3.74–8.05)	11.14 (3.56–24.02)	11.46 (3.56–29.75)	13.79 (4.42–45.88)	13.03 (10.64–17.49)	<0.01	38.4	60.0	0.01
PUSL1	4.53 (2.59–7.20)	9.01 (2.55–23.10)	11.60 (3.25–34.69)	11.53 (4.38–25.78)	11.85 (8.68–23.32)	<0.01	28.0	70.8	<0.01

## Discussion

Recent studies have shown that many patients with HCC whose disease exceeds BCLC staging recommendations for resection might benefit from hepatectomy [3–9]. However, in our previous studies 18.6% of these patients had unfavourable surgical outcomes, such as death within 1 year of surgery with tumour recurrence; therefore, these liver resections were regarded as futile procedures [10]. We believe that HCC is a highly heterogeneous tumour even when classified into the same clinical stage, and a thorough selection process is essential to ensure that only patients whose survival is likely to improve undergo liver resection.

Biomarkers are substances found in tissue, blood, or other body fluids that indicate diagnostic, prognostic, predictive, therapeutic, or other clinically relevant properties [25]. Many biomarkers for HCC and their corresponding targeted agents have been identified for use in the diagnosis and treatment of HCC [11–13]. In the present study, we aimed to find candidate biomarkers for which high expression was associated with higher BCLC staging and poorer OS of HCC patients, as they may reflect tumour characteristics indicative of prognosis (such as malignancy) and could thus be used to improve selection of candidates for surgery; patients with comparatively low expression of these biomarkers might have less malignant tumours and therefore might be likely to enjoy longer OS after hepatectomy. We found a total of 3315 HCC-related DEGs, among which 1568 genes were upregulated in HCC and were matched with probes in another dataset containing samples from 115 HCC patients with BCLC stage 0-C disease who underwent surgery. Using WGCNA, we identified 50 hub genes that were significantly correlated with BCLC staging.

The results of functional enrichment analysis of upregulated DEGs showed significant enrichment of genes involved in cell division and mitotic nuclear division, which might be related to proliferation of cancer cells. Pathway enrichment analysis showed that cell cycle, DNA replication, viral carcinogenesis, and RNA transport were significantly represented in the DEGs; these pathways are closely related to the occurrence and progression of tumours [26, 27]. However, functional enrichment analysis of 50 hub genes gave quite different results. The significant enrichment terms of hub genes were RNA processing, non-coding RNA processing and RNA phosphodiester bond hydrolysis, rather than the GO-BP terms related to cell proliferation identified for DEGs, such as positive regulation of cell division, mitotic nuclear division, and cell cycle. Furthermore, these hub genes showed no significant enrichments in biological pathways. We speculate that the hub gene products have complex molecular functions; that is advanced HCC reflects more than accelerated cell proliferation or enhanced inhibition of apoptosis, and may not result from alterations in only a few central molecular pathways.

Unsurprisingly, genes related to BCLC are also associated with HCC prognosis, as shown by the WGCNA results; in previous studies this may have been interpreted as a decline in patient prognosis as BCLC stage progresses [2]. To reassess hub gene expression levels associated with BCLC staging and screen out genes that significantly affect prognosis in HCC patients, we used UALCAN analysis. Among the 50 hub genes, 45 were identified from the TGCA HCC database; expression of 40% (18/45) significantly differed by tumour stage and 53% (24/45) were significantly associated with survival time of patients. Finally, we identified 13 hub genes that were closely related to both tumour stage and survival time: these were our candidate biomarkers.

Among these 13 hub genes, 4 (*C8ORF33*, *STIP1*, *Hsp90AB1* and *PRKDC*) have been implicated in HCC behaviour by previous studies [28–31]. *C8ORF33* has been associated with OS in HCC patients and could be used to distinguish poorly differentiated from well differentiated HCCs [28]. STIP1 acts as an adaptor protein that coordinates functions of HSP70 and HSP90 in protein folding, and as a secretory protein that regulates malignant cell growth. Autocrine STIP1 may inhibit HCC apoptosis through the PI3K–AKT-dependent pathway and is associated with poor prognosis in HCC [29]. Hsp90AB1 is frequently upregulated in cancer [30]. In the HCC cell line HepG2, HSP90AB1 expression is upregulated by hepatitis B virus encoded X protein (HBx), which may help reveal the role of HBx in hepatocarcinogenesis [31]. HSP90AB1 may also regulate angiogenesis, an important function in tumour progression [30]. *PRKDC* has been identified as critical to HCC pathogenesis and prognosis through RNA sequencing data [32]. Decreased PRKCA expression results in decreased cell proliferation, migration and invasion of HCC cells, suggesting a role for PRKDC in the malignant progression of HCC [33].

With regard to the other genes, *NUDCD1* (also known as *CML66* or *OVA66*) has been reported to have an oncogenic function in cervical carcinoma by favouring tumour cell proliferation, invasion, and metastasis associated with multiple pathways [34]. NUDCD1 is also expressed abundantly in primary acute myeloid leukaemia cells, acute lymphoid leukaemia cells, and chronic myelogenous leukaemia cells in advanced phase, compared with normal cells [35]. *INTS8* (also known as *C8orf52*) has been identified as a potentially mutational driver gene in endometrial carcinoma [36]; studies based on microarrays and exome-sequencing have proposed roles for INTS8 in gastric cancer [37] and peripheral T-cell lymphoma [38]. *DSCC1* is implicated in sister chromatid cohesion and DNA replication, and its elevated expression decreases apoptosis in colorectal cancer cells [39]. These data suggest that NUDCD1, INTS8, and DSCC1 might be novel indicators for HCC staging and prognosis, although the link between these three genes and HCC is currently unclear.

*TIGD* encodes POP1 (also known as ANXD2), which belongs to the Tigger subfamily of the Pogo superfamily of DNA-mediated transposons in humans. POP1 is a ribonuclease that localizes to the nucleus and affects pre-RNA processing. ARHGAP39 (also known as CrGAP or Vilse) is a Rho-GTPase activating protein that may affect dendritic structure and synaptic function in the brain [40]. The functions of *ZNF623*, *YDJC*, and *PUSL1* are unclear. However, their correlations with liver cancers warrant further study, as these genes and their products are potential prognostic markers of HCC.

## Conclusion

We identified 50 hub genes that correlated with BCLC staging of HCC patients, of which 13 hub genes (*TIGD5*, *C8ORF33*, *NUDCD1*, *INTS8*, *ZNF623*, *STIP1*, *HSP90AB1*, *DSCC1*, *POP1*, *ARHGAP39*, *PRKDC*, *YDJC* and *PUSL1*) also correlated with OS in HCC patients. We hypothesize that patients with BCLC stage B/C HCC and higher expression of these genes are likely to have poor prognoses after surgery, and curative surgical treatment might be avoided in such cases. Prospective studies are needed to confirm the gene functions and clarify the mechanisms of their effects on HCC staging and progression.

## Supporting information

S1 Table Gene expressions mapped with probes in GSE76427. Gene expressions mapped with probes in GSE76427 with pre-processed raw data.
(XLSX)

S2 Table External clinical traits of GSE76427. External clinical traits of GSE76427.
(XLSX)

S3 Table Gene significance and module membership based on WGCNA. Based on gene significance and module membership, the top 50 core genes in the Green module were regarded as hub genes.
(XLSX)
